# Gas Sensing Properties of p-Co_3_O_4_/n-TiO_2_ Nanotube Heterostructures

**DOI:** 10.3390/s18040956

**Published:** 2018-03-23

**Authors:** Onur Alev, Alp Kılıç, Çiğdem Çakırlar, Serkan Büyükköse, Zafer Ziya Öztürk

**Affiliations:** Department of Physics, Gebze Technical University, 41400 Kocaeli, Turkey; onuralev@gtu.edu.tr (O.A.); akilic@gtu.edu.tr (A.K.); ccakirlar@gtu.edu.tr (Ç.Ç.); sbuyukkose@gtu.edu.tr (S.B.)

**Keywords:** metal-oxide, gas sensor, nanostructures, heterostructure, nanotubes, TiO_2_, Co_3_O_4_

## Abstract

In this paper, we fabricated p-Co_3_O_4_/n-TiO_2_ heterostructures and investigated their gas sensing properties. The structural and morphological characterization were performed by scanning electron microscopy (SEM), X-ray diffraction (XRD), and X-ray photoelectron spectroscopy analysis (XPS). The electrical properties of the heterostructure were studied within the temperature range from 293 K to 423 K. Changes in electrical properties and sensing behavior against reducing and oxidizing gases were attributed to the formation of p–n heterojunctions at the Co_3_O_4_ and TiO_2_ interface. In comparison with sensing performed with pristine TiO_2_ nanotubes (NTs), a significant improvement in H_2_ sensing at 200 °C was observed, while the sensing response against NO_2_ decreased for the heterostructures. Additionally, a response against toluene gas, in contrast to pristine TiO_2_ NTs, appeared in the Co_3_O_4_/TiO_2_ heterostructure samples.

## 1. Introduction

Metal-oxide (MOX)-based gas sensors have a wide range of applications such as medical, air quality, energy efficiency, and the detection of hazardous gases. MOXs are preferred for sensing layers due to their easy fabrication, low cost, high sensor response, and easy integration [1]. Moreover, device performance is improved when sensors are fabricated with nanostructured MOXs. However, for almost all sensor applications, there is a strong demand for better sensor properties such as lower operation temperature and higher sensitivity and selectivity [2,3]. Different techniques, such as doping, loading, or heterostructure fabrication, have been employed to meet these requirements [4,5,6,7,8]. Among them, the combination of n-type and p-type MOX materials in the form of nanostructures is one of the most promising techniques [6,9]. An extended depletion layer between two different types of MOX semiconductors can provide higher sensor properties [9].

This study is focused on the combination of p-type cobalt oxide (Co_3_O_4_) and n-type TiO_2_. TiO_2_ is a very important material, especially for the sensing of H_2_ and VOCs, due to its high sensitivity and easy nanostructure fabrication [10,11,12,13,14,15]. Co_3_O_4_, well known as a catalytic activator in chemical processes such as oxidation reactions, has a great potential for sensor applications [16,17,18,19]. p-type Co_3_O_4_ can advance the sensing capabilities of TiO_2_ nanostructures. In this study, different amounts of Co_3_O_4_ were loaded on TiO_2_ nanotubes (NTs) to understand the heterostructure effect on sensing properties. The heterostructures were fabricated by a two-step electrochemical process. TiO_2_ NTs were fabricated via an anodization process and Co_3_O_4_ was loaded onto the NTs via electrochemical deposition. Then, electrical and sensor measurements were carried out.

## 2. Experiment

Co_3_O_4_/TiO_2_ heterostructures were fabricated via two-step electrochemical deposition consisting of anodization and cathodic deposition. Ti foils (20 mm in length and 10 mm in width) were provided from Sigma-Aldrich (St. Louis, MO, USA). NH_4_F (ammonium fluoride, 99.99%), ethylene glycol (99.8%), and cobalt (II) nitrate hexahydrate (99.999%) were purchased from Sigma-Aldrich. Ultra-pure (UP) water was used for all experiments. In the first step, Ti foils were cleaned with acetone, isopropanol, and UP water in ultrasonic bath, respectively. Then, the foils were dried in a nitrogen stream. An electrolyte solution was prepared with NH_4_F, ethylene glycol, and UP water with a molar ratio of 0.25:2:100. The anodization was carried out at 50 V with Pt foil as cathode in a thermos-stated bath at 20 °C for 1 h. Afterward, Co_3_O_4_ was loaded on TiO_2_ NTs (set as cathode) via cathodic deposition. The electrolyte for cathodic deposition was composed of 0.01 wt % cobalt (II) nitrate hexahydrate in UP water. Cathodic deposition was performed under three different conditions (5 V for 3 min, 10 V for 1 min, and 10 V for 3 min), and the resulting samples were named and are hereafter referred to as CT-1, CT-2, and CT-3, respectively. After the fabrication of Co_3_O_4_/TiO_2_ heterostructures, samples were rinsed with UP water and dried under a nitrogen stream. Finally, the fabricated samples were annealed at 500 °C for 3 h in an oxygen atmosphere to obtain a crystalline structure.

To investigate the crystal structure and morphological properties of the fabricated samples, XRD, SEM, and XPS methods were used.

**Figure 1:**
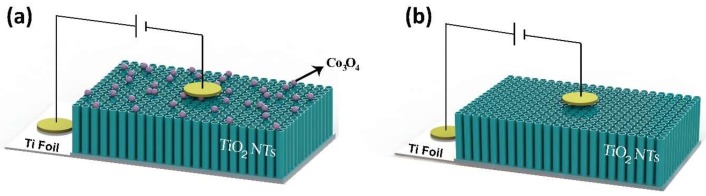
Measurement scheme of (**a**) pristine TiO_2_ nanotubes (NTs) and (**b**) Co_3_O_4_/TiO_2_ heterostructures.

Current–voltage (*I*–*V*) measurements were performed in the temperature range of 20–200 °C to investigate the electrical properties of fabricated samples. Au electrodes 100 nm thick were thermally evaporated on top of the fabricated samples as shown in Figure 1a,b. Because of the geometry of the devices, measurements were performed for vertical configuration (contacts are on top of the NTs and on Ti foil). A Keithley electrometer, model 6517 A, was used for electrical measurements with a Lakeshore 340 temperature controller. *I–V* curves were obtained by a linear voltage sweep between −1 V and +1 V with a scan rate of 0.05 V.

Pristine TiO_2_ and Co_3_O_4_/TiO_2_ heterostructure sensors were investigated toward H_2_, NO_2_, and VOCs (ethanol, acetone, toluene, and xylene). The sensors were mounted in a homemade test chamber with a volume of 1 L. Desired H_2_ and NO_2_ gas concentrations were obtained from gas tubes via mass flow controllers. VOCs were generated from cooled bubblers immersed in a thermal bath with dry air as a carrier gas [20]. Concentration of the VOCs were calculated using Antoine’s equation [21]. Baseline signal of the sensor was determined under dry air flow conditions. After the exposure to the target gases, recovery of the sensor from saturated conditions was achieved under dry airflow. Sensor measurements are presented in terms of sensor response, defined as *R_s_ = ΔI/I_0_*, where *ΔI* is the change in current. Here, *R_s_* is defined for reducing gases such as H_2_ and VOCs, and *ΔI* is given as *I_g_ − I_0_*. For oxidizing gases, such as NO_2_, sensor response is defined slightly differently: *R_s_ = ΔI/I_g_.* Here, *ΔI* is given as *I_0_ − I_g_*. For both formulations, *I_g_* is the current after the sensor is exposed to the reducing or oxidizing analyte gas, and *I_0_* is the reference value (baseline current) of the sensor exposed to high-purity dry air. 

## 3. Results and Discussion

Corresponding reactions for TiO_2_ and Co_3_O_4_ formation in the two-step electrodeposition route can be given as follows [17,22,23]:

(1a) $$2H_{2}O\to {\;O}_{2}+4e^{-}+4H^{+}$$

(1b) $$\mathrm{Ti}+O_{2}\to {\mathrm{TiO}}_{2}$$

(2a) $${\mathrm{CO}}_{3}O_{4}+H_{2}O\;+{\mathrm{OH}}^{-}↔3\mathrm{CoOOH}+e^{-}$$

(2b) $$\mathrm{CoOOH}+{\mathrm{OH}}^{-}↔{\mathrm{CoO}}_{2}+H_{2}O+e^{-}.$$

**Figure 2:**
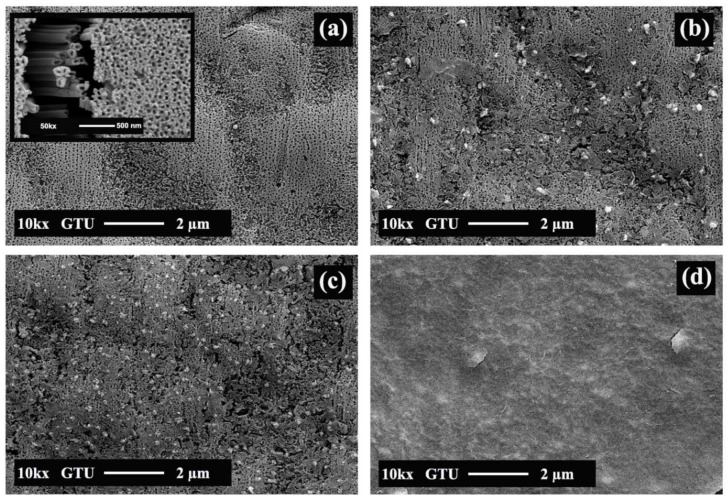
SEM images of (**a**) pristine TiO_2_ NTs, (**b**) CT-1, (**c**) CT-2, and (**d**) CT-3.

**Figure 3:**
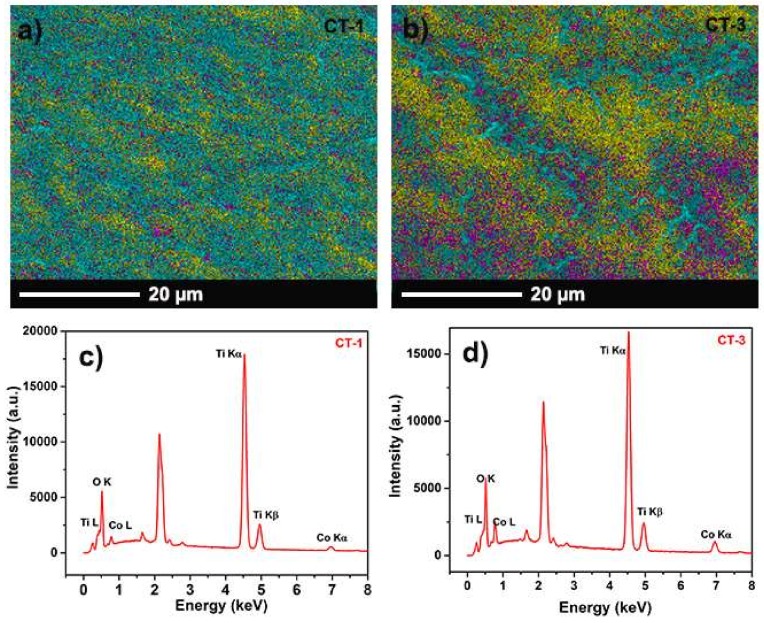
EDS mapping results of (**a**) CT-1 and (**b**) CT-3 sample, and EDS spectrum of (**c**) CT-1 and (**d**) CT-3 samples. In the mapping results, yellow represents O, blue represents Ti, and purple represents Co.

**Figure 4:**
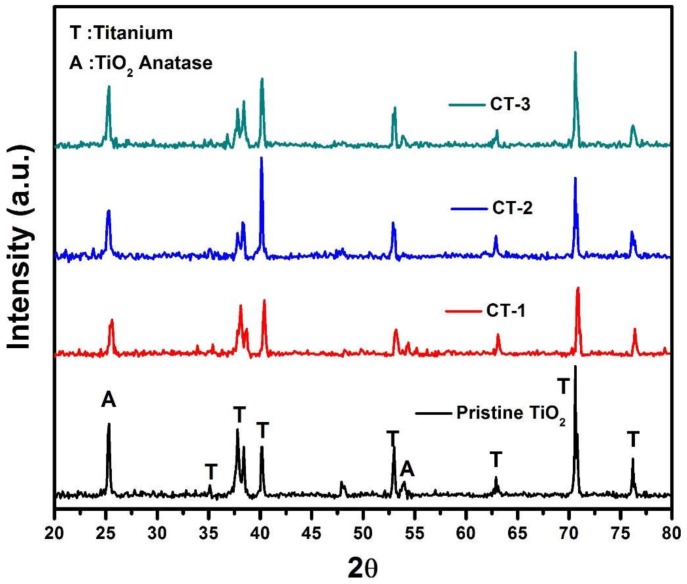
XRD patterns of pristine TiO_2_, CT-1, CT-2, and CT-3 samples.

Figure 2 shows SEM images of fabricated structures. Figure 2a indicates that a film of vertically aligned TiO_2_ NTs homogeneously covers the surface. The average NT diameter is 80 nm as seen in Figure 2a inset. Figure 2b–d shows cathodically deposited particles on the NTs. In the cathodic process, the amount of deposited Co_3_O_4_ changes with bias voltage and process time. Thin film formation was observed for CT-3, which was grown with a 10 V bias voltage and a 3 min process time (Figure 2d). EDS mapping and spectrums of CT-1 and CT-3 are given in Figure 3. The presence of the Co, Ti, and O was observed. XRD patterns of pristine TiO_2_ NTs and Co_3_O_4_/TiO_2_ heterostructures are given in Figure 4. The XRD patterns reveal that all diffraction peaks could be indexed to Ti and anatase TiO_2_ NTs [20]. According to the XRD results, Co_3_O_4_ particles on the surface most likely do not have crystalline structures. Even if there is a small degree of crystallinity for Co_3_O_4_ at 36.32°, the TiO_2_ reflection overlaps with that of the Co_3_O_4_, thus preventing any clearly visible peak from the rest of the signal [24].

**Figure 5:**
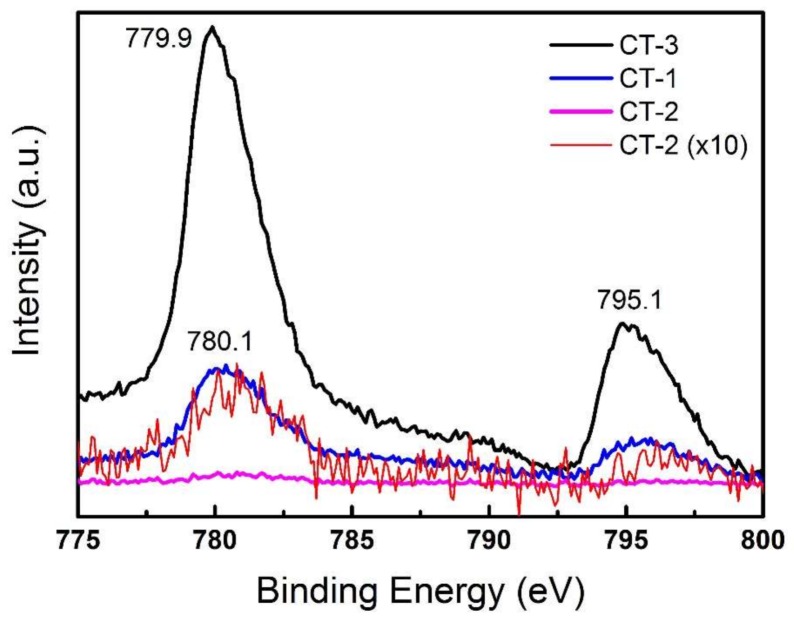
XPS spectra of Co 2p spectra of CT-1 (blue), CT-2 (red), and CT-3 (black). The intensity of CT-2 was magnified 10× (pink) for clarity.

To identify the surface chemical compositions and oxidation states of the NTs after Co deposition, X-ray photoelectron spectroscopy (XPS) was performed. CT-2 spectra shows Ti 2p and O 1s peaks originating from the TiO_2_ NTs. However, the Co 2p peak is very weak and can only be seen when the data is magnified (×10), which will be shown in the high-resolution XPS spectrum of the Co 2p region (Figure 5). This implies that the Co oxide coverage is very low in this sample. The presence of Co, in addition to the presence of Ti and O, is visible in the spectrum of CT-1, which indicates that the coverage is more pronounced in this sample. On the other hand, the CT-3 survey scan shows a more intense peak for Co 2p but not for Ti 2p, indicating that the surface is fully covered by cobalt oxide, which means that TiO_2_ NTs are too far under the surface to detect. SEM images of CT-1, CT-2, and CT-3 in Figure 2 support these findings. CT-1 and CT-2 show islands of Co_3_O_4_ on TiO_2_ NTs, whereas a full coverage is clear in CT-3.

Figure 5 shows Co 2p spectra of CT-1, CT-2 and CT-3. CT-1 and CT-2 spectra show two peaks at binding energies of 780.1 eV and 795.1 eV, corresponding to Co 2p3/2 and Co 2p1/2 peaks, implying a Co_3_O_4_ form [25]. The peak intensity of CT-2 is lower than CT-1 (the intensity of CT-2 is magnified 10× for clarity in Figure 4), indicating lower coverage compared to CT-1. The Co peak intensity of CT-3 is the highest, which was expected from the complete Co_3_O_4_ layer. The Co 2p3/2 peak of CT-3 shifted to 779.9 eV, which can be attributed to the increase in Co_3_O_4_ coverage from the sub-monolayer to the multilayer, resulting in a change in the local electronic properties and bonding geometries. Similar shifts have been observed in TiO_2_ and Al_2_O_3_ films as well [26,27,28].

**Figure 6:**
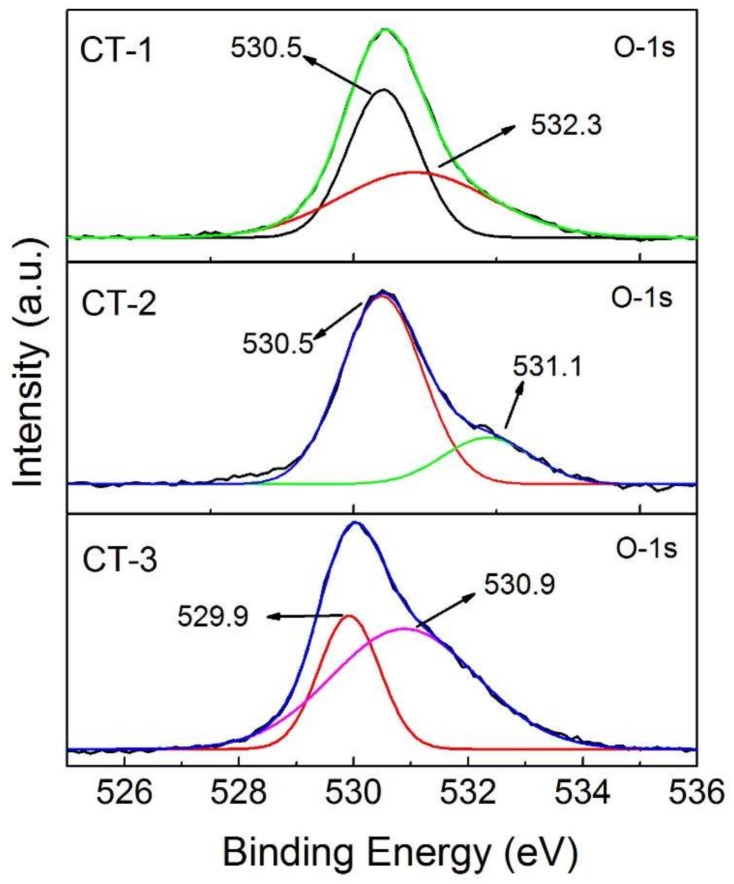
XPS spectra of the O 1s regions of CT-1, CT-2, and CT-3.

Figure 6 shows the high-resolution XPS spectrum of the O 1s regions for CT-1, CT-2, and CT-3, all of which can be fitted into two peaks. CT-1 and CT-2 show a primary peak at 530.5 eV, which can be attributed to the O–Ti bond in TiO_2_ [25]. The smaller secondary peaks at 532.3 eV and 531.1 eV in CT-2 and CT-1, respectively, can both be attributed to the O in Co_3_O_4_. In CT-1, the difference in peak height between the 530.5 eV peak and the secondary peak is higher compared to that in CT-2, suggesting that the Co_3_O_4_ contribution is less in this sample compared to CT-1, indicative of less Co_3_O_4_ coverage. In CT-3, where the surface is fully covered with Co_3_O_4_, the peak position of the Co–O bond is shifted from 531.1 eV to 529.9 eV and becomes the primary peak, possibly due to the Co_3_O_4_ coverage from the sub-monolayer to the multilayer. The small changes in the geometry of the film and the changes in the local electronic properties and bonding geometries could be the main contributor to the shift. Similar shifts have been observed for TiO_2_ and Al_2_O_3_ films as well [26,27,28]. The peak at 530.9 eV is attributed to the O–H group in the Co hydroxide precursor [25].

**Figure 7:**
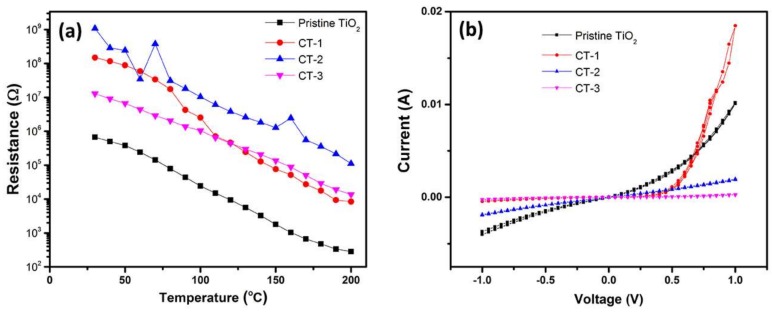
(**a**) Resistance vs. temperature of the sensors and (**b**) *I–V* curves at 200 °C.

Electrical characterization was performed under the condition and parameters as given in our previous work [29]. Electrical resistance of the heterostructures given in Figure 7a shows an increment with the loading of Co_3_O_4_. This behavior is attributed to the modification of the conduction channel by the depletion of charge carriers at the heterojunction interface. This mechanism, as discussed later in this text, is also responsible for the enhanced sensor performance of the heterostructures in terms of reducing gases. The *I–V* curves measured at 200 °C are given in Figure 7b. 

**Figure 8:**
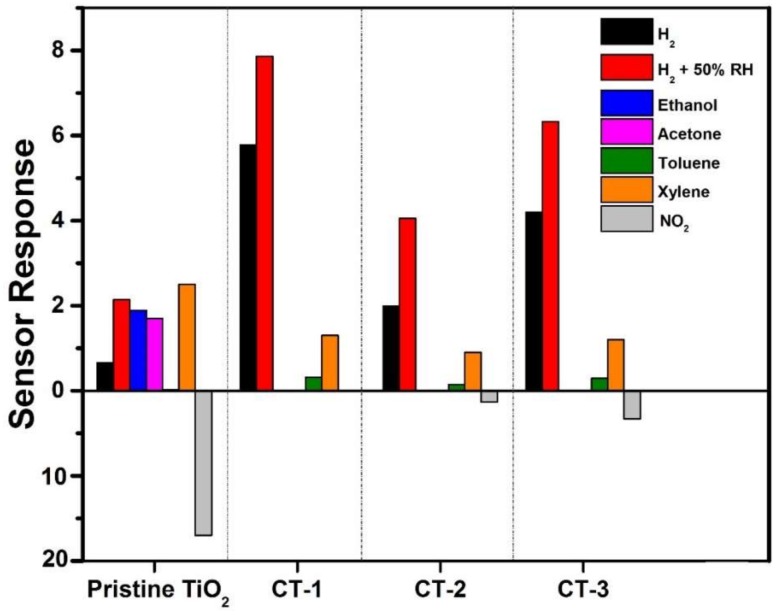
Sensor responses to 1000 ppm H_2_ and VOCs and to 50 ppm NO_2_ at an operation temperature of 200 °C.

Amperometric sensor measurements of H_2_, VOCs (ethanol, acetone, toluene, xylene, and chloroform), and NO_2_ were performed under a constant voltage at different operation temperatures. The gas sensing responses of samples at 200 °C are shown in Figure 8. These data confirm the absence of sensitivity toward acetone and ethanol for Co_3_O_4_/TiO_2_ heterostructures, while TiO_2_ NTs could sense both VOCs. On the other hand, the sensor response to hydrogen increased with Co_3_O_4_/TiO_2_ heterostructures. The CT-1 sample, compared to the sample of pristine TiO_2_ NTs, showed an approximately nine-fold higher response to H_2_. In addition, the response to toluene starts to appear after Co_3_O_4_ loading. Sensor responses to 1000 ppm H_2_ in 50% relative humidity (RT) can be seen in Figure 8. It is well known that the presence of humidity can cause a deterioration in sensor properties [30]. Despite the relative magnitudes of heterostructure sensor responses, compared with those of pristine TiO_2_, to pure H_2_ and H_2_/humidity mixtures, there was no significant improvement observed when humidity was eliminated (Figure 8). In addition to H_2_ and VOCs, sensors responses to 50 ppm NO_2_ are given in Figure 7. The sensor responses are 17, 0.02, 1.32, and 3.3 for pristine TiO_2_, CT-1, CT-2, and CT-3, respectively. 

**Figure 9:**
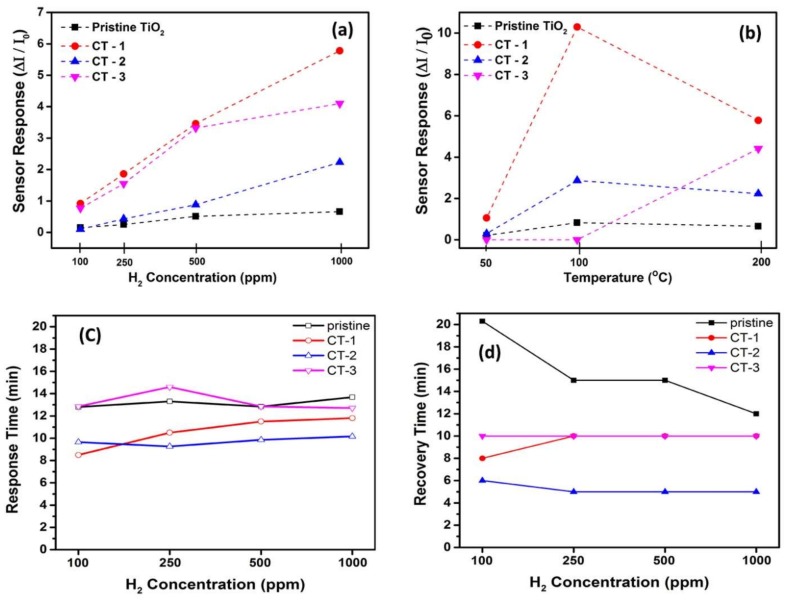
(**a**) Sensor response vs. H_2_ concentration at 200 °C. (**b**) Sensor response vs. operation temperature for 1000 ppm H_2_. (**c**) Response time vs. H_2_ concentration at 200 °C. (**d**) Recovery time vs. H_2_ concentration at 200 °C.

**Figure 10:**
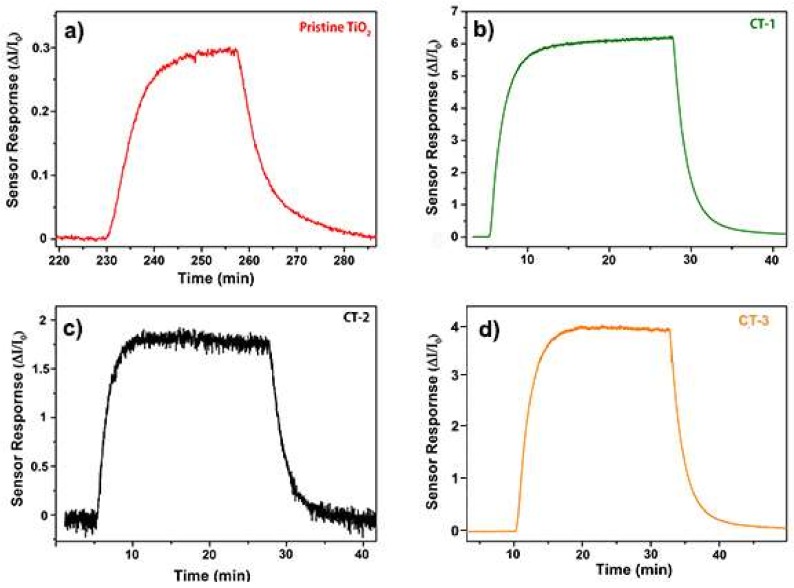
Sensor response versus time graphs of (**a**) pristine TiO_2_, (**b**) CT-1, (**c**) CT-2, and (**d**) CT-3 to 1000 ppm H_2_ at 200 °C.

Figure 9a shows sensor responses for different H_2_ concentrations at 200 °C. Co_3_O_4_-loaded samples could detect lower H_2_ concentrations than pristine TiO_2_ could. The CT-1 sample exhibited the highest sensor responses at every concentration of H_2_. Sensor response vs. temperature graph was given in Figure 9b. The CT-1 sample showed better sensor responses at all temperatures. Although the CT-1 sample showed the highest sensor response at 100 °C, a better response time and recovery time for CT-1 were observed at 200 °C (data not shown). Dynamic sensor responses can be seen in Figure 10. It is obvious that, to obtain the best sensor performance from these devices, operation temperature is a critical variable, and its optimum value will be different for each device. Regarding the response time and recovery time, the loaded samples exhibited lower values and thus better performance than did the pristine TiO_2_ NTs, as can be seen in Figure 9c,d.

Based on the above results, Co_3_O_4_-loaded TiO_2_ NTs heterostructures showed enhanced sensor properties compared to the pristine TiO_2_ NT sensor. Sensing mechanisms of MOX sensors that are composed of only p-type or n-type materials have been studied and well explained in the literature [31,32,33,34,35]. Enhanced sensor properties can be attributed to variation in the resistance by the formation of the p–n junction [35]. Even though there is no readily accepted and developed mechanism with detailed sensing treatment for heterostructures, two considerations might be taken into account as responsible mechanisms. The first one is the catalytic effect of Co_3_O_4_. In this case, Co_3_O_4_ plays a role as a catalyst material in the reaction between analyte gas and TiO_2_ [36]. If a continuous film is formed on the surface, a catalytic role of the Co_3_O_4_ turns into a sensing layer, so a lower sensor performance generally is observed [37]. This idea is also supported by the sensing behavior of the CT-3 sensor response (in Figure 9) and by the XPS and SEM data that indicate full coverage on the surface for CT-3. Previous works have reported the enhanced sensor properties due to the catalyst role and hydrogen sensitivity of Co_3_O_4_ [16,38,39].

Another possible mechanism that is responsible for the enhanced performance of the sensors is the formation of the p–n junction at the interface between p-type Co_3_O_4_ and n-type TiO_2_ [31,39]. This formation is partially observed on electrical measurements with an increase in the resistance with Co_3_O_4_ loading (Figure 7). Especially, the CT-1 sensor that exhibits optimal sensor properties evidently showed diode-like rectifying behavior. Due to the depletion region at the heterojunction interface, which causes a narrower cross-sectional area for charge carriers in the nanorod, an increase in resistance is observed. A reduced cross-sectional area available for charge conduction in the nanowire will result in increased resistance. When reducing gases such as VOCs or H_2_ are introduced, gas molecules can diffuse into the interface, modify the junction, and thus enhance the sensor response by decreasing the initially high resistance value because of the released electrons into the junction [5]. A relatively easy diffusion of H_2_ molecules into the interface, appears as sensing selectivity against H_2_ in Figure 8 [40]. Contrary to reducing gases, introduction of an oxidizing gas such as NO_2_ will give an additional increase in resistance due to a further widening of the depletion region. However, a tendency for the resistance to further increase will not appear noticeably in sensor response due to the presence of exceedingly high resistance. This mechanism well explains the difference in response data of our samples with respect to reducing and oxidizing gases, given in Figure 8. Another important parameter of the electrical behavior of the p–n junction is operation temperature because of its role in carrier concentration. Its effect appears prominently in Figure 9b.

## 4. Conclusions

p-Co_3_O_4_/n-TiO_2_ heterostructures were fabricated via a two-step electrochemical process involving anodization and cathodic deposition. The structure and morphology of the heterostructures were characterized by SEM, XRD, and XPS, which confirms that Co_3_O_4_ particles were spread uniformly over the TiO_2_ NT surface. Changes in electrical resistance and *I–V* curves partially support the p–n junction formation. The p-Co_3_O_4_/n-TiO_2_ heterostructures, in comparison with those of the pristine TiO_2_ NTs, show significantly improved H_2_ sensing response at 200 °C, which is an up to 9-fold increase. Co_3_O_4_ loading also provides a sensing response against toluene. In contrast, sensing response against NO_2_ decreased for Co_3_O_4_-loaded samples. This difference in sensor behavior against reducing and oxidizing gases might be attributed to the formation of p–n heterojunctions, leading to the formation of a carrier depletion region and modification of the electrical characteristics of the device with an enhancement in gas sensor characteristics.
